# A Chemo-Ecological Investigation of *Dendrilla antarctica* Topsent, 1905: Identification of Deceptionin and the Effects of Heat Stress and Predation Pressure on Its Terpene Profiles

**DOI:** 10.3390/md21090499

**Published:** 2023-09-19

**Authors:** Paula De Castro-Fernández, Carlos Angulo-Preckler, Cristina García-Aljaro, Conxita Avila, Adele Cutignano

**Affiliations:** 1 Department of Evolutionary Biology, Ecology and Environmental Sciences (BEECA), Faculty of Biology, Universitat de Barcelona (UB), 08028 Barcelona, Catalonia, Spain; conxita.avila@ub.edu; 2 Institut de Recerca de la Biodiversitat (IRBio), Universitat de Barcelona (UB), 08028 Barcelona, Catalonia, Spain; 3 Department of Genetics, Microbiology and Statistics, Faculty of Biology, Universitat de Barcelona (UB), 08028 Barcelona, Catalonia, Spain; crgarcia@ub.edu; 4 Consiglio Nazionale delle Ricerche (CNR), Istituto di Chimica Biomolecolare (ICB), 80078 Pozzuoli, Napoli, Italy; acutignano@icb.cnr.it; 5 Red Sea Research Center and Computational Bioscience Research Center, King Abdullah University of Science and Technology (KAUST), Thuwal 23955-6900, Saudi Arabia; carlos.preckler@kaust.edu.sa

**Keywords:** marine benthic invertebrates, Antarctic benthos, natural products, diterpenoids, chemical ecology, sponges, global change

## Abstract

Marine sponges usually host a wide array of secondary metabolites that play crucial roles in their biological interactions. The factors that influence the intraspecific variability in the metabolic profile of organisms, their production or ecological function remain generally unknown. Understanding this may help predict changes in biological relationships due to environmental variations as a consequence of climate change. The sponge *Dendrilla antarctica* is common in shallow rocky bottoms of the Antarctic Peninsula and is known to produce diterpenes that are supposed to have defensive roles. Here we used GC-MS to determine the major diterpenes in two populations of *D. antarctica* from two islands, Livingston and Deception Island (South Shetland Islands). To assess the potential effect of heat stress, we exposed the sponge in aquaria to a control temperature (similar to local), heat stress (five degrees higher) and extreme heat stress (ten degrees higher). To test for defence induction by predation pressure, we exposed the sponges to the sea star *Odontaster validus* and the amphipod *Cheirimedon femoratus*. Seven major diterpenes were isolated and identified from the samples. While six of them were already reported in the literature, we identified one new aplysulphurane derivative that was more abundant in the samples from Deception Island, so we named it deceptionin (**7**). The samples were separated in the PCA space according to the island of collection, with 9,11-dihydrogracilin A (**1**) being more abundant in the samples from Livingston, and deceptionin (**7**) in the samples from Deception. We found a slight effect of heat stress on the diterpene profiles of *D. antarctica*, with tetrahydroaplysulphurin-1 (**6**) and the gracilane norditerpene **2** being more abundant in the group exposed to heat stress. Predation pressure did not seem to influence the metabolite production. Further research on the bioactivity of *D. antarctica* secondary metabolites, and their responses to environmental changes will help better understand the functioning and fate of the Antarctic benthos.

## 1. Introduction

Antarctic shallow rocky bottom communities are sponge-rich associations of organisms with a complex network of biological interactions [1]. In these communities, sponges provide habitat and food for many other species, being a fundamental element of these benthic ecosystems [2,3,4,5]. Many of the species living in these communities use chemical communication and therefore are rich in natural products [6,7,8,9,10,11,12]. As with other organisms, sponges produce biologically active natural compounds to avoid predation, competition, pathogenic infections, or fouling, among other things [13,14].

*D. antarctica* Topsent, 1905, is a common demosponge inhabiting shallow waters in the coastal areas of Antarctica [15]. Its distribution area comprises the Antarctic Peninsula but also southern South America, the Falkland Islands, the subantarctic and other locations in Antarctica (including McMurdo Sound, the Wilheim II Coast, Victoria Land and the Graham Coast) from depths 10–549 m [16,17]. It usually forms large and massive yellow carpets covering rocks and other surfaces, sometimes reaching a few meters in size. Its surface is bright in colour and presents conspicuous spiky conules. It usually adopts a lobular or an encrusting shape. *D. antarctica* forms three dimensional structures where other organisms may also live [18]. It may also grow on top of other organisms (macroalgae or other invertebrates).

Its chemical defences, mostly diterpenoids, provide the sponge with protection to avoid predation [12,19,20,21,22,23,24,25]. Potential predators for the species include sympatric generalist predators like the sea star *O. validus*, omnivorous amphipods, or the common nudibranch *Doris kerguelenensis* [26,27]. A recent study has shown that abundances of amphipod communities living near the sponges are related to the metabolic profile of the sponge [18].

The role of chemical ecology is pivotal in these communities [28] and its relevance for sponges has become clear over recent years [18,22,29,30]. For *D. antarctica*, the defensive role of its metabolites represents an ecological advantage that may be affected by global change, since increasing water temperatures may affect, for example, the production of these chemicals and thus the sponge’s protection against potential predators. Similarly, changes in predation pressures may also produce changes in these natural products [31]. Some of the sponge compounds can display relevant bioactivities in the laboratory too [19,24,25,32,33,34]. *D. antarctica* compounds have been described as having antibacterial, antifungal, or cytotoxic activity, among others. Both 9,11-dihydrogracilin A and membranolide showed activity against *B. subtilis* [19], whereas membranolide and darwinolide inhibited methicillin resistant *S. aureus* biofilm [25]. The compounds 9,11-dihydrogracilin A and 9,11-dihydrogracillinone A were found to have antifouling activity [22]. Tetrahydroaplysulphurin-1 displayed low micromolar activity against the *Leishmania* parasite *L. donovani* [33]. Ciaglia and co-workers suggested that 9,11-dihydrogracilin A exerts anti-inflammatory effects and has anti-edema activity in vivo [35]. Besides the terpenes, *D. antarctica* is also known to produce bioactive alkaloids, such as the yellow pigment 4,5,8 trihydroxyquinoline-2-carboxylic acid, which inhibits the growth of some marine bacteria [36], as well as picolinic acid and 7-methyladenine, suggested to play a defensive role [23].

Sponges have abundant and common associations with a wide range of microorganisms, forming what is called a “holobiont” [37,38,39,40]. The sponge microbiota may play a role in either the biological production or compound variability reported in Antarctic species [4,41,42,43,44]. Their species composition is affected by geographic, environmental and host factors. This is relevant for the role they play within the sponge [45,46]. The microbiota of *D. antarctica* has recently been studied [42,47].

In this study we aimed at (1) analysing the profile of secondary metabolites in populations of the sponge *D. antarctica* from Livingston and Deception Islands (South Shetland Island), and (2) assessing the effects of heat stress and predation pressure on the chemical profile of the sponge. The identification and structural characterization of a new terpene derivative from specimens collected on Deception Island is also presented.

## 2. Results

### 2.1. Major Diterpenes in D. antarctica Populations from Two Different Localities

**Figure 1:**
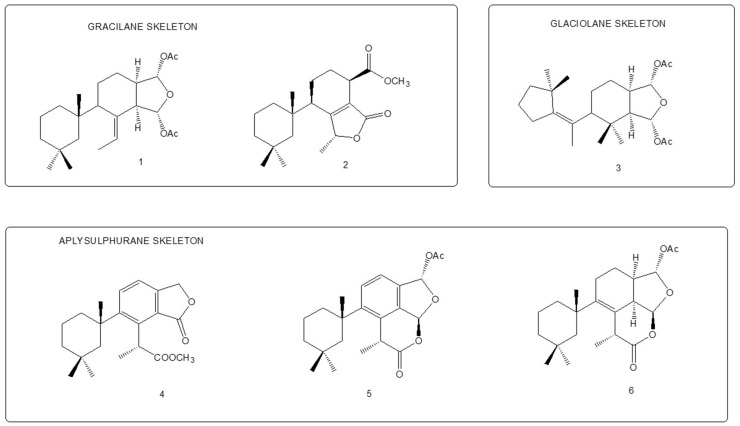
Diterpene metabolites identified in *D. antarctica* in this study.

**Figure 2:**
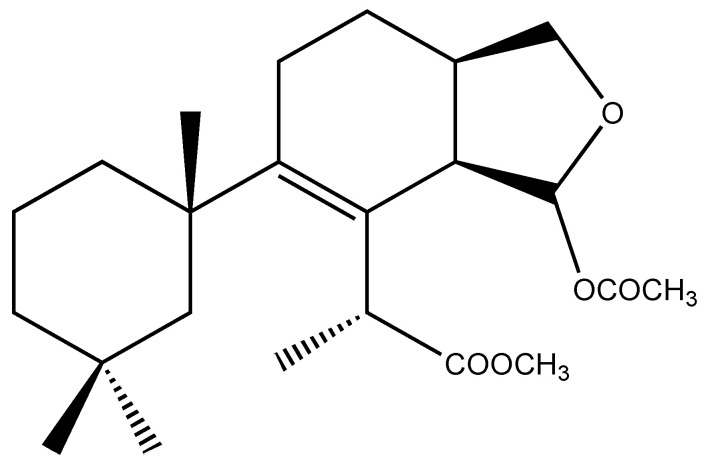
Chemical structure of deceptionin (**7**).

We analysed the chemical profile of *D. antarctica* specimens collected from Deception (*n* = 5) and Livingston (*n* = 13) Islands, in the South Shetland Archipelago, Antarctica. Data from gas chromatography-mass spectrometry (GC-MS) revealed seven most prominent peaks in the extracts, putatively identified as terpenes. The identity of six of them was confirmed after their purification from the raw extract as 9,11-dihydrogracilin A (**1**) [19] (Rt = 26.1) and the gracilane norditerpene **2** [48] (Rt = 26.2), the glaciolane norditerpene **3** [49] (Rt = 28.0), and three aplysulphurane derivatives, membranolide (**4**) [19] (Rt = 29.3), aplysulphurin (**5**) [50] (Rt = 35.3) and tetrahydroaplysulphurin-1 (**6**) [51] (Rt = 35.6) (Figure 1 and Supplementary Materials Figures S1–S6). The spectroscopic data of the remaining compound did not match with any of the known terpenes and was characterized as a new aplysulphurane derivative especially abundant in the specimens from Deception Island, which therefore we named deceptionin (Rt = 20.3) (**7**, Figure 2 and Supplementary Materials Figures S7–S15).

### 2.2. Structural Characterization of Deceptionin *(**7**)*

**Figure 3:**
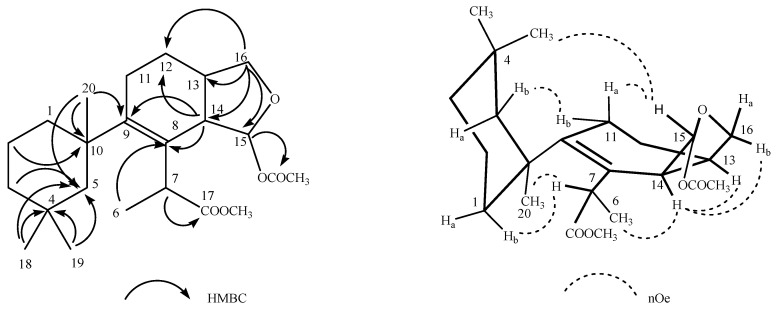
Key HMBC and NOE correlations observed for deceptionin (**7**).

Deceptionin (**7**) gave a molecular ion adduct [M + Na]^+^ in HRESIMS at *m*/*z* 415.24591 which was consistent with the molecular formula C_23_H_36_O_5_ (theor. 415.24550) requiring six degrees of unsaturation (Figure S8). The ^13^C NMR spectrum (Figure S10) showed four resonances attributable to sp^2^ carbons (Table 1). In particular, quaternary carbon signals at 128.8 and 146.0 ppm accounted for a tetra-substituted non conjugated double bond (C-8/C-9) while two downfield shifted signals at 174.9 and 170.6 ppm were assigned to ester carbonyl groups, in agreement with the stretching band observed in the FT-IR spectrum at 1739.48 cm^−1^. In particular, the molecular ion peak [M − 60]^+^ observed in the ESIMS/MS spectrum suggested the presence of an acetyl group, while a deep interpretation of the GCMS spectrum allowed to infer the occurrence of a methoxycarbonyl function due to consecutive losses of MeOH and CO from the M-Me-AcOH fragment at *m*/*z* 317.23 (Figure S7). Hence, the three remaining formal unsaturations had to be assigned to cycles. A diagnostic methine carbon at 103.5 ppm (C-15), bearing a proton resonating as doublet at *δ* 5.87 (*J* = 2.5 Hz) in the ^1^H NMR spectrum (Figure S9), was indicative of an acetal function which fulfilled the number of oxygens required by the molecular formula. Starting from this latter proton, COSY and TOCSY correlations allowed to easily depict the spin system of the bicyclic substructure including a cyclohexene ring fused with a cyclic acetal (Figure 2). In fact, the acetal proton (H-15) was coupled to an allylic proton at *δ* 2.91 (dd, *J* = 8.1, 2.5 Hz, H-14) in turn coupled with a methine at *δ* 2.41 (H-13) (Figures S11–S12). This latter signal showed correlations in the COSY spectrum with one of the two protons of the oxygenated methylene C-16 at δ 4.09 (dd, *J* = 8.6, 5.6 Hz) and the proton signal at *δ* 1.26 of the methylene at C-12. The methylene H_2_-12 was coupled with the allylic methylene at C-11; thus, with the support of HSQC and HMBC data (Figures S13–S14) the planar structure of the bicyclic system was unambiguously assigned (Figure 3). The linkage with the remaining cycle was secured by the key HMBC correlation of the singlet methyl resonance at *δ* 1.08 (H_3_-20) with the olefinic carbon at 146.0 ppm (C-9) through the quaternary carbon at 41.4 ppm. The cyclohexane ring bearing two geminal methyl groups at *δ* 0.88 and 0.89 on C-4 was easily assigned by combining homo and heteronuclear correlation data. Finally, a bis-allylic methine quartet at *δ* 4.22 (*J* = 6.9 Hz, H-7) coupled to a methyl group at *δ* 1.24 (H_3_-6), connected the methoxycarbonyl function to the olefinic carbon at 127.6 ppm (C-8), thus completing the planar structural elucidation of the aplysulphurane skeleton of the new diterpene molecule **7** (Figure 3). The relative configuration of C-7 and C-10 was assumed to be the same as that reported for known aplysuphurane derivatives, whereas the stereochemistry of the bicyclic system was proposed by inspecting NOE experiments. In particular, the observed NOE between the methine protons H-14 and H-13 suggested a *cis* junction of the two cycles and the correlation of H-14 with H_b_-16 indicated that they all lay on the same face of the molecular plane. On the other hand, the acetal proton H-15 showed NOE effects with both the methyl group H_3_-18 and the H-11a proton that was consistent with the orientation depicted in Figure 3 (Figure S15). A literature survey revealed that deceptionin NMR signals were similar to those of the methyl ester of pourewic acid A [52] (Table 1) from which it mainly differed in having an acetoxy instead of a methoxy acetal function at C-15. Interestingly, a compound with the same planar structure of pourewic acid A but opposite in sign optical rotation was reported by another research group [48] and the two compounds were suggested to be enantiomers [53]. NOE data recorded for deceptionin were consistent with those reported in [48], suggesting the relative configuration as depicted in Figure 2.

### 2.3. Terpene Profile of Sponges in Their Natural Habitat

**Figure 4:**
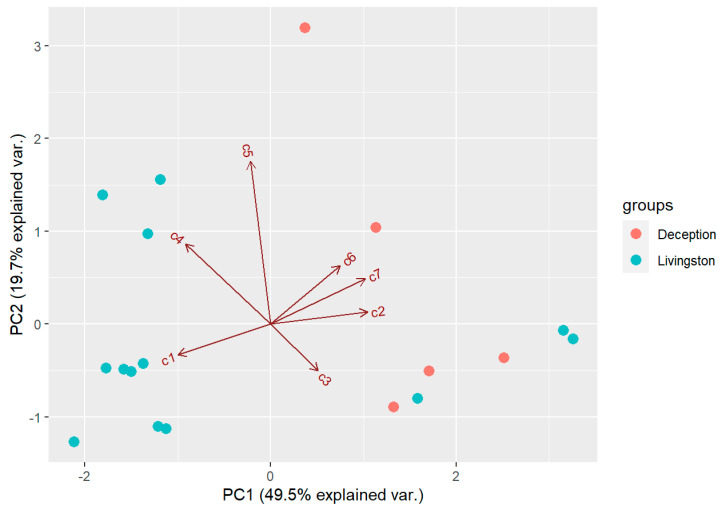
Biplot space of the first two principal components extracted in the PCA of the samples from the natural habitat. Deception = samples from Deception Island, Livingston = samples from Livingston Island. c1–c7=compounds **1**–**7** analyzed.

The results indicate that there is a high variability in the chemical profiles of the *D. antarctica* specimens analysed, as observed in standard deviation values for natural habitat samples (Table 2). PCA extracted three components with an eigenvalue >1, which explained 83.6% of the overall variability in the metabolite profiles. The first component (PC1) explained 49.5% of the overall variability in the metabolite profiles and mostly opposed the abundance of **7** and **2** (positive values) to that of **1** and **4** (negative values). The second component (PC2) explained 19.7% of the overall variability in the metabolite profiles and was influenced by the abundance of **5** (positive value). The third component (PC3) explained 14.4% of the overall variability in the metabolite profiles and was influenced by the abundance of **1** and **3** (positive values). PCA revealed that the sponge specimens separated in the biplot space according to their island of origin. In general, sponge specimens from Deception Island were enriched in **7** and **2**, whereas those from Livingston Island were enriched in **1** and **4** (Figure 4). There were three specimens from Livingston Island that grouped with Deception Island sponges, and one specimen from Deception Island that was enriched in **5** and depleted in **3**. PERMANOVA revealed the chemical profiles of the two groups of sponges were significantly different (*p* < 0.05) between islands. Univariate tests revealed that the concentrations of **1** and **7** were significantly different between the two groups of sponges (Mann–Whitney–Wilcoxon test; *p* < 0.05). Also, total terpene concentration was significantly higher on average in samples from Livingston (6.00 ± 3.45 mg/g DW) than in samples from Deception Island (2.51 ± 0.83 mg/g DW) (*p* < 0.05).

### 2.4. Chemotyping

**Figure 5:**
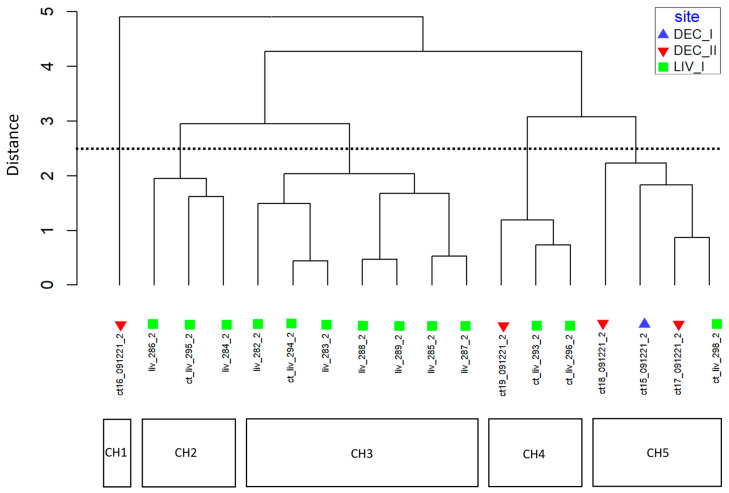
Chemotype clustering of the natural habitat samples. CH = chemotype. DEC-I, DEC-II = sampling sites in Deception Island. LIV = sampling site in Livingston Island.

**Figure 6:**
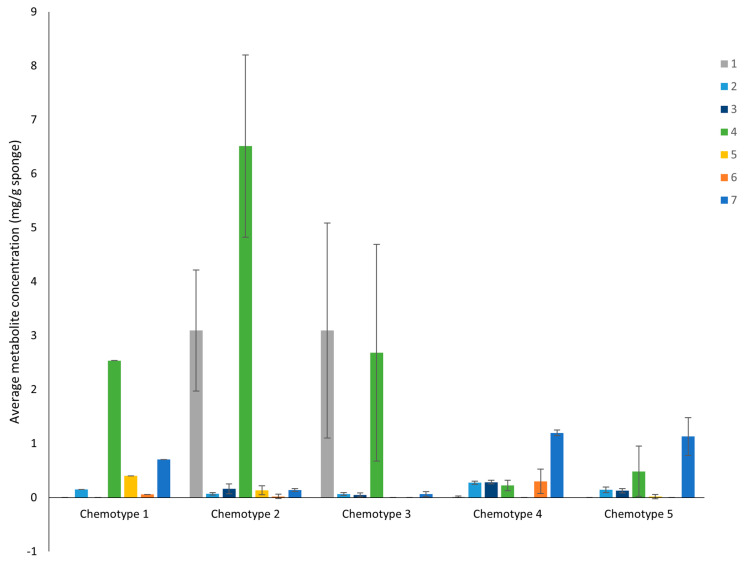
Average concentration of the seven most abundant diterpenes **1**–**7** from the natural habitat *D. antarctica* for each chemotype.

**Figure 7:**
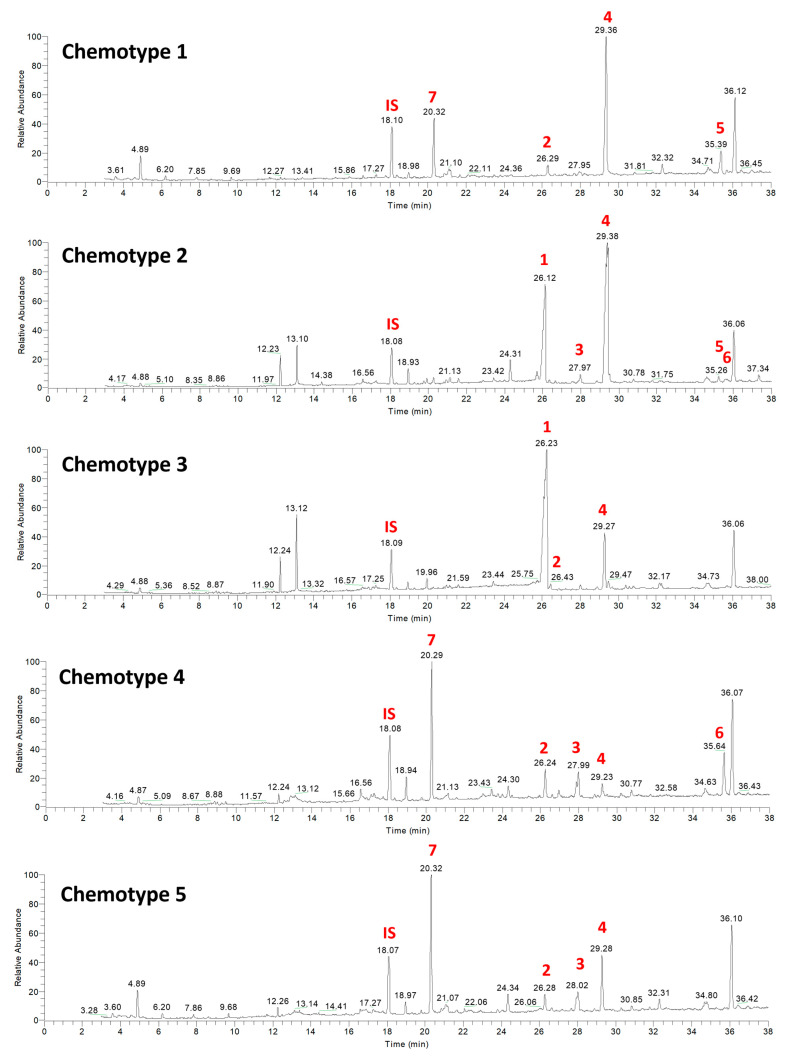
Representative chemical profiles of each chemotype for the natural habitat samples of *D. antarctica*. Main terpene derivatives **1**–**7** are reported. IS = internal standard.

The relative abundance and dominance of the seven major metabolites within individual samples indicates that there are chemically distinct phenotypes within the *D. antarctica* populations of Deception and Livingston Islands. The samples were clustered according to the abundance of the metabolites and five chemotypes were identified in the natural habitat sponges (Figure 5). All Livingston Island samples but three were clustered into two different chemotypes (chemotypes 2 and 3). Chemotype 2 was characterized by the dominance of **4** and, to a lesser extent, **1**, with trace metabolites **5** and **6**. Chemotype 3 was characterized by the dominance of **1** over all other metabolites (Figure 6). The other three Livingston samples clustered with Deception Island samples in chemotypes 4 and 5. Chemotypes 1, 4 and 5 were characterized by higher abundance of **7**, only a minor terpene in chemotypes 2 and 3. One sample from Deception conformed with chemotype 1, since it had only **2**, **4**, **5** and **7** and in different proportions than the other samples. The main difference between chemotypes 4 and 5 is the presence of **6** in chemotype 4, absent in chemotype 5 (Figure 7).

### 2.5. Effect of Experimental Heat Stress on the Secondary Metabolites of D. antarctica

**Figure 8:**
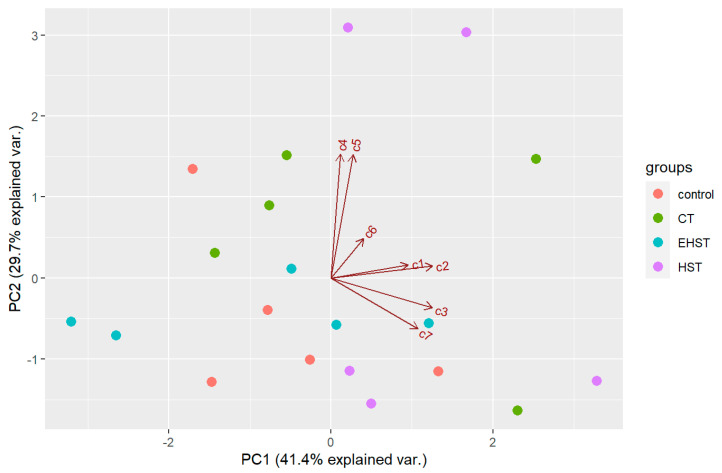
Biplot space of the PCA of the samples from the heat stress experiment. Control = natural habitat samples (Deception Island), CT = control temperature, HST = heat stress temperature, EHST = extreme heat stress temperature. c1–c7 = compounds **1**–**7** analysed.

There was an effect of the temperature in the natural products of *D. antarctica*. PCA extracted two components with an eigenvalue >1, which explained 71.1% of the overall variability in the metabolite profiles (Figure 8). The first component (PC1) explained 41.4% of the overall variability in the metabolites profiles and was influenced by the abundance of **2** and **3** (positive values). The second component (PC2) explained 29.7% of the overall variability in the metabolites profiles and was influenced by the abundance of **4** and **5** (positive values). The samples did not seem to follow any pattern in the biplot space, and PERMANOVA revealed that the chemical profile was not significantly different between the experimental groups (*p* > 0.05). Univariate ANOVAs revealed no significant difference in the concentration of any of the seven compounds between the experimental groups (*p* > 0.05). Pair-wise tests of each compound revealed a significant difference in the concentration of **2** and **6** between the control and the HST group (*p* < 0.05). Also, total terpene concentration was higher on average in the HST group (4.36 ± 2.14 mg/g DW) than in the natural habitat group (2.51 ± 0.83 mg/g DW), although the difference was not statistically significant (*p* > 0.05).

### 2.6. Effect of Experimental Predation Pressure on the Secondary Metabolites of D. antarctica

**Figure 9:**
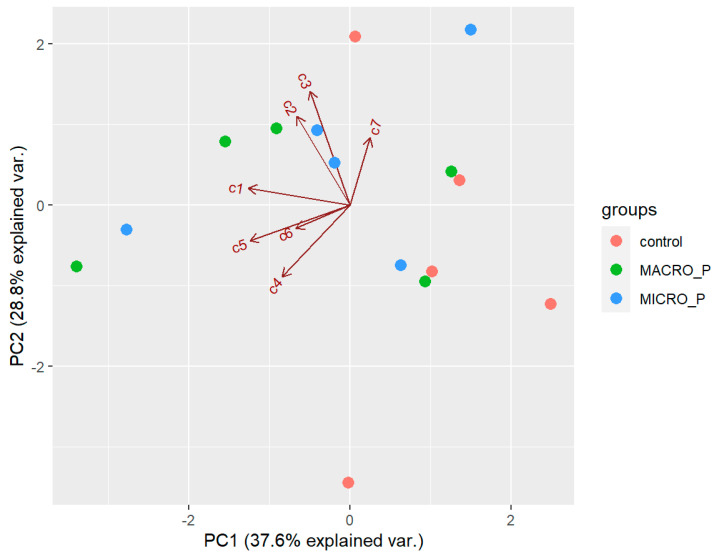
Biplot space of the first two principal components extracted in the PCA of the sample from the predation pressure experiment. Control = natural habitat samples (Deception Island), MACRO_P = samples exposed to macro-predation, MICRO-P = samples exposed to micro-predation. c1–c7 = compounds **1**–**7** analyzed.

There was no effect of predation on the natural products of *D. antarctica*. PCA extracted three components with an eigenvalue >1, which explained 81.4% of the overall variability in the metabolite profiles. The first component (PC1) explained 37.6% of the overall variability in the metabolite profiles and was influenced by the low abundance of **1** and **5** (negative values). The second component (PC2) explained 28.8% of the overall variability in the metabolites profiles and was influenced by **2** and **3** (positive values). The third component (PC3) explained 15% of the overall variability in the metabolites profiles and mostly opposed the abundance of **4** (positive value) to that of **6** (negative value). The samples did not seem to follow any pattern in the biplot space (Figure 9), and PERMANOVA revealed that the chemical profile was not significantly different between the experimental groups (*p* > 0.05). Univariate ANOVAs revealed no significant differences in the concentration of any of the seven compounds between the experimental groups (*p* > 0.05). Pair-wise tests of each compound neither revealed significant difference in the concentration of the individual compounds between the control and the macro-predation group, nor between the control and the micro-predation group (*p* > 0.05). However, total terpene concentration was higher on average in the macro-predation samples (3.57 ± 0.98 mg/g DW) than in the natural habitat group (2.51 ± 0.83 mg/g DW), although the difference was not statistically significant (*p* = 0.088).

### 2.7. Antimicrobial Assays

Membranolide (**4**) showed antimicrobial activity against *E. coli* O157:H7 at 200 μg, presenting a zone of inhibition of 10 mm in the two replicates performed. Following Mahon and co-workers’ criteria, this could be considered as a “strong” (+++) growth inhibition [54]. The other tested compounds did not show any activity against the selected bacterial strains.

## 3. Discussion

Benthic invertebrates in general, and sponges in particular, are a rich source of bioactive molecules that could serve as base for developing new drugs or have an industrial application. The samples of *D. antarctica* contained seven major diterpenes, one of which was characterized as a new aplysulphurane derivative, deceptionin (**7**). Further antimicrobial assays using different pathogenic strains, as well as cytotoxicity and other bioactivity tests should be performed to search for the potential bioactivity of the compound.

The diterpene profile was highly variable among the samples, even in the samples collected from the same sampling site. Not only all seven metabolites were not present at all the samples, but also the concentrations and proportions among compounds varied. This variability in the chemical profile between individuals of the same species is not uncommon [55,56,57] and was already reported for *D. antarctica* populations around Palmer Station (Anvers Island) [18]. Von Salm and co-workers also found high variability in the terpene concentration even between replicates from the same sampling site [18]. Despite the high interindividual variability, in general the samples grouped according to the island of origin. There were two main differences in the diterpene profile between the sponges from Livingston and Deception Island. One of them was the considerably higher concentration of total terpenes in the sponges from Livingston Island than in the sponges from Deception Island. The second one was the concentration of two of the major diterpenes: 9,11-dihydrogracilin A (**1**), more abundant in Livingston Island samples, and deceptionin (**7**), more abundant in Deception Island samples, while almost absent in Livingston Island samples. 

Although both collection sites are shallow rocky bottoms harbouring relatively similar benthic communities, there are environmental factors that may be playing a role in *D. antarctica* chemical profile: the waters of Port Foster (the inner bay and caldera of the volcanic Deception Island) have higher water temperatures [58], a presence of suspended volcaniclastic particles [59] and chemicals from local geothermal activity [60,61]. We could expect that sponges exposed to higher environmental pressures, presumably those from Deception Island, would have higher concentrations of defensive compounds [13]. However, regarding total terpene concentration, we observed significantly higher concentrations of compounds in Livingston than in Deception Island. This could perhaps be related to a higher predation pressure in Livingston Island, which should be further analysed. The higher presence of deceptionin (**7**) in *D. antarctica* from Deception Island could be a response to a particular stress characteristic of the environment of the coasts of Deception Island. 

The populations of *D. antarctica* analysed so far are not chemically homogenous around the continent. In McMurdo Sound, *D. antarctica* was reported to yield 9,11-dihydrogracilin A (**1**) and membranolide (**4**) [19], and also dendrillin in a later study [23]. The ecological role of 9,11-dihydrogracilin A (**1**) has not been reported yet. It has been suggested to play a role against predators found in areas not dominated by macroalgae and thus with less abundance of amphipods [62]. Specimens from Terra Nova Bay instead presented only 9,11-dihydrogracilin (**1**) and dendrinolide [20]. Populations from around Palmer Station (Western Antarctic Peninsula) have been studied several times, with reports including aplysulphurin (**5**), tetrahydroaplysulphurin-1 (**6**) and membranolide (**4**) as the major diterpenoids [18,32], another study including also darwinolide [25], and a more recent analysis identifying up to eleven diterpene derivatives, the major constituents being 9,11-dihydrogracilin A (**1**), membranolide (**4**), aplysulphurin (**5**) and tetrahydroaplysulphurin-1 (**6**), and the minor constituents being glaciolide, the norditerpene (**3)**, cadlinolide C and dendrillins A–D [24]. To our knowledge, just one study considered the effect of location on the chemical diversity of *D. antarctica*, but they found no significant effect. Sampling locations were all within a small area around Palmer Station [18]. From the evidence so far, we could suggest that there is not a single diterpenoid derivative found in every studied population of the sponge. However, it seems that some compounds are found in the two main regions studied (the Ross Sea and the Western Antarctic Peninsula), like 9,11-dihydrogracilin A (**1**) and membranolide (**4**), while aplysulphurin (**5**), tetrahydroaplysulphurin-1 (**6**) and darwinolide are only found in *D. antarctica* from the Antarctic Peninsula, and dendrinolide was only reported in McMurdo Sound (Ross Sea). However, further studies on the influence of location on the chemical profile of the sponge should be conducted. Also, the same populations collected around Palmer Station (Anvers Island, Western Antarctic Peninsula) showed differential diterpenoid profiles depending on the year of collection [33]. 

Metabolic and genetic studies of some Antarctic benthic species, such as the rhodophyte *Plocamium cartilagineum* [63,64] and the nudibranch *D. kerguelenensis* [65] have shown significant metabolic variation between specimens mostly corresponding to different phylogroups. Cycles of glaciation have isolated regions, allowing independent divergence among individuals of the same species, which could lead to speciation, as shown for *D. kerguelenensis* [66]. High genetic connectivity and subsequent homogeneity was found for *D. antarctica* populations from different locations off the South Shetland Islands and Northern Antarctic Peninsula [67]. However, signs of local adaptation were found for the samples of Deception Island, showing activation of genes mostly involved in immune and stress responses. This was associated with the physicochemical particularities of the island as an active volcano, with higher temperatures and distinct environmental conditions from the surrounding islands. Specimens from Livingston Island were not analysed in this genetic study.

Significant differences between *D. antarctica* collections or populations led von Salm and co-workers to hypothesize that selective predation pressures may be driving its chemical diversity [18]. These authors found different diterpene profiles in *D. antarctica* in the different habitats where they lived: shallower zones dominated by canopy-forming macroalgae and with an abundance of omnivorous amphipods harboured *D. antarctica* that had a greater concentration of tetrahydroaplysulphurin-1 (**6**) than those collected from deeper, more exposed habitats depleted in amphipods. In our study, the collection sites in both islands are similar shallow rocky bottoms dominated by similar species. However, Deception Island is a very particular place with peculiar shallow water communities [68]. The bottoms of this island are covered by volcanic ashes, with a huge abundance of echinoderms, and with some rocky areas fully covered by filter-feeding organisms [69]. This particular environment may have driven the selection and evolution of the natural metabolites production abilities present there.

Methanolic extracts of *D. antarctica* showed chemotactic food response in the spongivorous sea star *Perknaster fuscus* [23,70] and lipophilic extracts showed deterrence on the omnivorous amphipod *Gondogeneia antarctica* in feeding deterrence assays [26]. However, which molecules are responsible for this activity is yet to be discovered. Membranolide (**4**) deterred the amphipod *G. antarctica* [71] but was not found in higher concentrations in the sponges from the high amphipod abundance habitat [18]. In our study, we did not find any significant pattern of the effect of micro-predation (the amphipod *C. femoratus*) on the diterpenoids profile of *D. antarctica*. Similarly, we did not find an effect of macro-predation (the seastar *O. validus*). Exposure to predator species that may not elicit an antipredatory activity may have hindered the effect of predation pressures. Maybe the exposure to predator species that are known to be deterred by compounds produced by the sponge may elicit the chemical response of the sponge. Also, perhaps using exclusively individuals with the same chemotype would help better ascertain the potential effect of the factors tested on the chemistry of the sponge. 

Although we did not find any significant changes in the chemical profile of sponges exposed to predation pressure, tetrahydroaplysulphurin-1 (**6**) and the gracilane norditerpene (**2**) were significantly more abundant in the samples exposed to a heat stress (around 5 °C above the local seawater temperature) than in the natural habitat samples from Deception Island. 

Terpenes and terpenoids are a large class of natural products commonly regarded as of fungal and plant origin whose biosynthesis by bacteria is attracting increasing research interest [72]. Considering the broad distribution of terpene/terpenoid synthase genes across bacterial genomes [72], it is tempting to argue that terpenoid biosynthesis in marine sponges could be mediated by bacterial symbionts, emerging as a further mechanism possibly conferring host defence against natural enemies or mediating microbe–microbe interactions within the sponge host.

Further characterisation of the chemical profile in *D. antarctica* from different sites should be carried out to keep gaining insight into the factors affecting or regulating the chemical ecology in benthic organisms. Further experiments with QGI, a larger number of samples, and/or testing specimens with the same chemotype or clones would allow better observation of the effect of these stressors, that clearly pose a risk to Antarctic communities in the frame of the current global change.

## 4. Materials and Methods

### 4.1. Sample Collection

**Figure 10:**
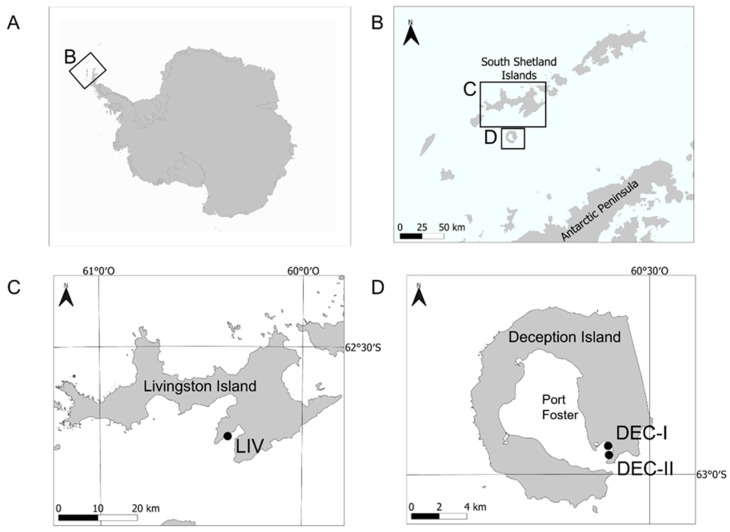
Location of the sampling sites. (**A**) Antarctic continent. (**B**) Tip of the Antarctic Peninsula and South Shetland Islands. (**C**) Livingston Island (South Shetland Islands). (**D**) Deception Island (South Shetland Islands). Sampling stations are marked with dots.

Healthy whole specimens of *D. antarctica* were collected by hand using scuba from different stations in Livingston (*n* = 13) and Deception (*n* = 30) Islands (South Shetland Islands, Antarctica) (Figure 10). Additionally, bulk *D. antarctica* specimens were collected for the extraction of chemical standards needed for quantification in study specimens. Collection sites were shallow (depths of 20–25 m) rocky bottoms. Collection took place during January 2018 and January 2019. Sponge specimens were kept in plastic containers and transported to the lab within less than 1 h. There, some specimens were directly frozen at −20 °C and some others were used for the aquaria experiments. 

### 4.2. Heat Stress and Predation Pressure Experiments in Aquaria

To measure the effect of the heat stress in the production of metabolites, 15 specimens of *D. antarctica* were placed in aquaria at three different temperatures, including local seawater temperature, to be used as control temperature (CT), 0.5 ± 0.3 °C (mean ± SD temperature along the experiment), and at two higher temperatures: heat stress temperature (HST), 5.4 ± 0.4 °C, and extreme heat stress temperature (EHST), 9.7 ± 0.8 °C. The temperatures were chosen according to the increase predicted by the IPCC and other reports, by duplicating the expected values and higher, and to the aquarium possibilities available for us to carry out the experiments. Water temperature was measured and controlled using a digital controller (Aqua Medic T controller twin) connected to heating (Sera 50 W or 150 W) and/or cooling (Aqua Medic Titan 150) units.

To measure the effect of the predation pressure in the production of metabolites, five specimens of *D. antarctica* were placed in an aquarium at local seawater temperature with two specimens per sponge of the red sea star *O. validus*, an omnivorous predator with circumpolar distribution. In a separate tank, five specimens of *D. antarctica* were placed along with ~200 specimens per sponge of the amphipod *C. femoratus*, a spongivorous species very common in these shallow benthic assemblages [73].

For both experiments, the system was kept steady throughout the experiment. Sponge specimens were kept in compartmented tanks (volumes ranging between 24–112.5 L according to the organism sizes), with seawater circulating through all the compartments, and were incubated for ca. three weeks.

### 4.3. General Analysis

Optical rotation was measured on a Jasco P-2000 digital polarimeter at 589 nm (Jasco, Milan, Italy). FT-IR spectrum was recorded on a Jasco FT/IR 4100 spectrophotometer. The UV spectrum was acquired on a Jasco V-650 Spectrophotometer. One-dimensional and two-dimensional NMR spectra were recorded on a Bruker AVANCE™ III HD-400, equipped with a CryoProbe™ Prodigy or on a Bruker DRX-600 equipped with TXI CryoProbe^TM^ (Bruker, Milan, Italy) in CDCl_3_ (*δ*_H_ values reported refer to CHCl_3_ protons at 7.26; *δ*_C_ values refer to CDCl_3_ carbon at 77.0 ppm). High resolution mass spectra were acquired on a Q-Exactive Hybrid Quadrupole-Orbitrap Mass Spectrometer (Thermo Scientific, Milan, Italy). GC-MS analyses were performed on an ion-trap MS instrument in EI mode (70 eV) (Polaris Q, Thermo Scientific) connected with a GC system (GCQ, Thermo Scientific) by a 5% phenyl/methyl polysiloxane column (30 m × 0.25 mm × 0.25 µm, VF-5 ms, Agilent Technologies, Cernusco sul Naviglio (MI), Italy) using helium as a gas carrier. HPLC analyses were performed on a Shimadzu high-performance liquid chromatography system (Shimadzu, Milan, Italy) equipped with binary LC-20AD pumps in line with a Diode Array Detector SPD-M20A. TLC plates (KieselGel 60 F254) and silica gel powder (Kieselgel 60 0.063–0.200 mm) were from Merck (Milan, Italy). Chemicals were of analytical reagent grade and solvents of HPLC/LCMS grade (Merck) and were used without any further purification.

### 4.4. Metabolomic Analysis

Ca. 100 mg of each freeze-dried study specimen were extracted with dichloromethane (DCM) (3 × 10 mL). Phytyl acetate (80 μg) was added as an internal standard before the first extraction. Combined extracts were concentrated under N_2_ stream and redissolved in DCM (500) μL for GC-MS analysis. The following temperature gradient was applied: initial 160 °C holding for 3 min, then increase of 3 °C min^−1^ up to 260 °C followed by 30 °C min^−1^ up to 310 °C, holding for 3 min at 310 °C; split flow 10 mL min^−1^; Transfer line T = 280 °C; Inlet T = 290 °C; Ion source T = 250 °C; full scan *m*/*z* 50–500. Injection of 2 μL analytical runs were processed by using Xcalibur software (vers. 2.2 SP1.48) (Thermo-Scientific). All samples were analysed in duplicate.

### 4.5. Isolation and Characterization of Diterpenes from D. antarctica

Three freeze-dried specimens of *D. antarctica* collected on Livingston Island, were pooled together (35.6 g) and extracted with methanol (MeOH) (6 × 400 mL); the raw extract (16.1 g) was redissolved in MeOH/H_2_O 9:1 and partitioned with *n*-hexane (4 × 100 mL) following a modified Kupchan method [74]. The *n*-hexane extract (1.9 g) was fractionated by silica chromatography on column eluted with a gradient of solvents from 100% petroleum ether (PE) to 100% diethyl ether (EE). Fraction 19l eluting with PE/EE 9:1 afforded pure 9,11-dihydrogracilin A (**1**) (10.1 mg).

Twelve freeze-dried specimens collected in Deception Island were pooled together (30 g) and extracted with DCM (6 × 300 mL); the raw extract (1.4 g) was fractionated by silica chromatography on column with gradient elution from 100% PE to 100% EE. The fractions eluted by PE/EE 9:1 were further fractionated by normal phase-high performance liquid chromatography (NP-HPLC) on a Luna Silica column (250 × 4.6 mm, 5 μm) (Phenomenex, Castel Maggiore (BO), Italy), flow rate 1 mL min^−1^, with PDA detector monitoring at λ 210, 220, 260 and 272 nm; for the elution *n*-hexane (A) and *n*-hexane:isopropanol (97:3) (B) were used applying an isocratic method: initial 60% A and 40% B holding for 30 min. Peak collected at Rt 8 min was analysed by MS and NMR and identified as **3** (0.7 mg) by comparison with data from the literature.

The fractions eluted by PE/EE 1:1 were further fractionated by semipreparative NP-HPLC (Kromasil KR100-5-Sil column, Merck, 250 × 10 mm, 5 μm) flow rate 3.5 mL min^−1^) with PDA detector monitoring at λ 210, 220, 260 and 272 nm; for the elution *n*-hexane (A) and *n*-hexane:isopropanol (97:3) (B) were used applying the following gradient: initial 60% A and 40% B holding for 35 min, followed by increase to 100% B in 15 min, then returning to original conditions in 1 min; a re-equilibration time of 9 min was included between runs. Peaks collected at Rt 25, 31 and 42 min were analysed by MS and NMR and identified as **2** (0.6 mg), tetrahydroaplysulphurin-1 (**6**) (0.4 mg) and membranolide (**4**) (1.2 mg) by comparison with data from the literature.

The fractions eluted by PE/EE 8:2 were further purified by silica chromatography on column and then fractioned by semipreparative NP-HPLC (Kromasil KR100-5-Sil column, 250 × 10 mm, 5 μm, flow rate 3.5 mL min^−1^) with PDA detector monitoring at λ 210, 220, 260 and 272 nm; for the elution *n*-hexane (A) and *n*-hexane:isopropanol (97:3) (B) were used applying the following gradient: initial 80% A and 20% B, then increase to 30% B in 35 min, followed by increase to 65% B in 7.5 min, then returning to original conditions in 1 min; a re-equilibration time of 6.5 min was included between runs. Peaks collected at Rt 15 and 20 min were analysed by MS and NMR and identified as aplysulphurin (**5**) (4.1 mg) and deceptionin (**7**) (1.9 mg) by comparison with data from the literature.

### 4.6. Deceptionin *(**7**)*

[α]_D_ −7.38° (c 0.05, MeOH); IR (film) ν_max_ = 2929, 2851, 1739, 1561, 1454, 1367, 1234, 1084, 1006, 933 cm^−1^; UV λ_max_ (ε) =206 (3409); HRESIMS^+^: *m*/*z* 415.24591 [M + Na]^+^ accounting for C_23_H_36_O_5_Na^+^ (theor. 415.24550). For NMR data see Table 1.

### 4.7. Data Analysis

The diterpenes were quantified using the internal standard phytyl acetate. The amount of each metabolite was calculated by peak area normalization to the IS area and expressed as mg/g sponge dry weight (DW). Transformation of the data using fourth root was applied to the natural habitat samples subset to achieve homoscedasticity. Untransformed data was used for the heat stress and predation pressure experimental samples. Each subset of samples was standardized. Euclidean matrix distance was generated after a fourth root transformation was applied to the natural habitat data set, and a dendrogram was plotted with samples clustered using the complete method. Principal components analysis and permutational analysis of variance (PERMANOVA) were performed for the three subsets of samples: natural habitat samples, heat stress experiment samples, and predation pressure experiment samples. Analysis of variance (ANOVA) was performed for each compound on the three subsets of samples. Wilcoxon pair-wise tests were used for comparisons between the control group and the experimental group.

Statistical tests were performed using R (version 4.2.0).

### 4.8. Antimicrobial Assays

The Kirby–Bauer disk diffusion susceptibility test was used to assess the potential antimicrobial activity of some of the isolated diterpenoids of *D. antarctica*. The metabolites 9,11-dihydrogracilin A (**1**), membranolide (**4**), aplysulphurin (**5**), tetrahydroaplysulphurin-1 (**6**), and deceptionin (**7**) were tested against the human pathogens *E. coli* O157:H7 (ATCC 43888) and *S. aureus* (ATCC 9144). Two hundred μg of each compound were loaded onto 6-mm paper disks in a volume of 20 μL of chloroform, which saturated the disk. Pure cultures of the bacterial strains were grown on TSA plates for 24 h. Then, isolated colonies were suspended using a sterile cotton swab in a NaCl 0.85% solution until reaching the turbidity of a McFarland 0.5 standard. A spread culture in Mueller–Hinton agar plates was prepared from this solution using a sterile cotton swab. Disks were placed on the seeded plates and plates were incubated at 37 °C. A chloramphenicol disk (10 μg) was used as a positive control. An unloaded disk and a disk loaded only with 20 μL of the solvent were used as negative controls. The test was performed in duplicate. After 24 h, activity was checked observing the halo of growth inhibition around the disks. When there was a halo, the diameter was measured. Growth inhibition activity was classified following Mahon and co-workers’ criteria [54].

## Figures and Tables

**Table 1:** ^1^H and ^13^C NMR data of deceptionin (**7**). (400 MHz, CDCl_3_). Spectroscopic data of methyl pourewate A are reported as a comparison from reference [52].

Position	Deceptionin (7)		Methyl Pourewate A	
	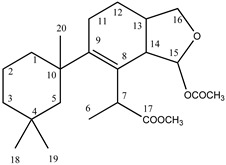		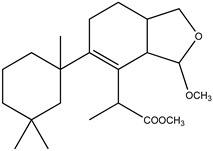	
	^1^H, *δ*, mult, *J* (Hz)	^13^C, ppm	^1^H, *δ*, mult, *J* (Hz)	^13^C, ppm
1a 1b	1.29, m; 2.09, m	39.1	1.26, m; 2.18, m	39.2
2a 2b	1.49, m; 1.80, m	20.1	1.49, m; 1.94, m	20.0
3a 3b	1.21, m; 1.33, m	39.9	1.18, m; 1.38, m	40.1
4	-	31.6	-	31.6
5a 5b	1.06, m; 1.77, d, 13.5	50.7	0.97, m; 1.84, m	50.9
6	1.24, d, 6.9	15.9	1.20, d, 6.8	16.1
7	4.22, q, 6.9	41.9	4.18, q, 4.4	42.0
8	-	127.6	-	128.2
9	-	146.0	-	143.9
10	-	41.4	-	41.6
11a 11b	1.94, ddd, 16.0, 14.0, 4.2; 2.23, ddd, 16.0, 4.2, 4.2	27.4	1.86, m; 2.20, m	27.7
12a 12b	1.26, m; 1.64, dq, 13.0, 4.2	30.2	1.24, m; 1.65, m	31.2
13	2.41, m	37.8	2.34, q, 7.6	38.0
14	2.91, dd, 8.1, 2.5	47.1	2.65, dd, 8.1, 2.4	49.0
15	5.87, d, 2.5	103.5	4.62, d, 2.4	110.8
16a 16b	3.85, d, 8.6; 4.09, dd, 8.6, 5.6	76.0	3.76, dd, 8.8, 3.4; 4.02, dd, 8.5, 6.4	74.9
17	-	174.9	-	174.7
18	0.89, s	27.1	0.92, s	26.6
19	0.88, s	32.6	0.88, s	33.3
20	1.08, s	30.4	1.02, s	30.9
O*CO*CH_3_	-	170.6		
OCO*CH*_3_	2.04, s	21.5		
OCH_3_ (methyl ester)	3.59, s	51.6	3.67, s	51.7
OCH_3_ (methoxy)			3.24, s	54.6

**Table 2:** Concentration values of the main terpene derivatives **1**–**7** in *D. antarctica* specimens. Values are represented as mean ± SD (mg/g DW). CT = control temperature, HST = heat stress temperature, EHST = extreme heat stress temperature.

	1	2	3	4	5	6	7
**Natural habitat**							
Deception Island	0.00 ± 0.01	0.16 ± 0.07	0.14 ± 0.12	0.92 ± 0.98	0.10 ± 0.18	0.01 ± 0.03	1.16 ± 0.30
Livingston Island	2.38 ± 2.01	0.11 ± 0.09	0.12 ± 0.10	2.99 ± 2.76	0.03 ± 0.07	0.07 ± 0.16	0.30 ± 0.42
**Heat stress experiment**							
CT	0.02 ± 0.03	0.20 ± 0.07	0.20 ± 0.11	1.44 ± 0.85	0.55 ± 0.38	0.15 ± 0.14	1.04 ± 0.53
HST	0.01 ± 0.01	0.27 ± 0.09	0.20 ± 0.09	1.29 ± 1.53	0.67 ± 0.92	0.35 ± 0.24	1.57 ± 0.49
EHST	0.00 ± 0.01	0.14 ± 0.11	0.12 ± 0.10	0.45 ± 0.39	0.01 ± 0.02	0.41 ± 0.54	0.79 ± 0.62
**Predation experiment**							
Macropredation	0.04 ± 0.04	0.21 ± 0.04	0.19 ± 0.06	1.34 ± 0.51	0.35 ± 0.40	0.12 ± 0.18	1.32 ± 0.15
Micropredation	0.02 ± 0.03	0.22 ± 0.04	0.21 ± 0.04	0.81 ± 0.84	0.21 ± 0.34	0.22 ± 0.28	1.28 ± 0.43

## Data Availability

The data presented in this study are available on request from the corresponding author.

## Supplementary Materials

The following supporting information can be downloaded at: https://www.mdpi.com/article/10.3390/md21090499/s1, Figure S1. EIMS spectrum of 9,11-dihydrogracilin A (**1**); Figure S2. EIMS spectrum of compound **2**; Figure S3. EIMS spectrum of compound **3**; Figure S4. EIMS spectrum of membranolide (**4**); Figure S5. EIMS spectrum of aplysulphurin-1 (**5**); Figure S6. EIMS spectrum of tetrahydroaplysulphurin-1 (**6**); Figure S7. EIMS spectrum of deceptionin (**7**); Figure S8. HRESI-MS of deceptionin (**7**); Figure S9. ^1^H NMR spectrum (400 MHz, CDCl_3_) of deceptionin (**7**); Figure S10. ^13^C NMR spectrum (100 MHz, CDCl_3_) of deceptionin (**7**); Figure S11. ^1^H, ^1^H COSY NMR spectrum (400 MHz, CDCl_3_) of deceptionin (**7**); Figure S12. ^1^H, ^1^H TOCSY NMR spectrum (400 MHz, CDCl_3_) of deceptionin (**7**); Figure S13. ^1^H, ^13^C edHSQC NMR spectrum (400 MHz, CDCl_3_) of deceptionin (**7**); Figure S14. ^1^H, ^13^C HMBC NMR spectrum (400 MHz, CDCl_3_) of deceptionin (**7**); Figure S15. ^1^H, ^1^H NOESY NMR spectrum (400 MHz, CDCl_3_) of deceptionin (**7**).
