# Supplementary material for: A Chemo-Ecological Investigation of Dendrilla antarctica Topsent, 1905: Identification of Deceptionin and the Effects of Heat Stress and Predation Pressure on Its Terpene Profiles
Source: Mar Drugs. 2023 Sep 19;21(9):499. doi: 10.3390/md21090499 (PMC10532619; doi:10.3390/md21090499)
Supplement: Supplementary file 1 [file marinedrugs-21-00499-s001.zip › marinedrugs-2559429-supplementary.pdf]

Supplementary Material

A Chemo-Ecological Investigation of *Dendrilla antarctica* Topsent, 1905: Identification of Deceptionin and the Effects of Heat Stress and Predation Pressure on Its Terpene Profiles

Paula De Castro-Fernández[1,2,3,4,*], Carlos Angulo-Preckler[5], Cristina García-Aljaro[3], Conxita Avila[1,2], and Adele Cutignano[4]

Figure S1. EIMS spectrum of 9,11-dihydrogracilin A (**1**).

Figure S2. EIMS spectrum of compound **2**.

Figure S3. EIMS spectrum of compound **3.**

Figure S4. EIMS spectrum of membranolide (**4**).

Figure S5. EIMS spectrum of aplysulphurin (**5**)**.**

Figure S6. EIMS spectrum of tetrahydroaplysulphurin-1 (**6**)

Figure S7. EIMS spectrum of deceptionin (**7**).

Figure S8. HRESI-MS of deceptionin (**7**).

Figure S9. ^{1}H NMR spectrum (400 MHz, $CDCl_3$) of deceptionin (**7**).

Figure S10. ^{13}C NMR spectrum (100 MHz, $CDCl_3$) of deceptionin (**7**).

Figure S11. ^{1}H, ^{1}H COSY NMR spectrum (400 MHz, $CDCl_3$) of deceptionin (**7**).

Figure S12. ^{1}H, ^{1}H TOCSY NMR spectrum (400 MHz, $CDCl_3$) of deceptionin (**7**).

Figure S13. ^{1}H, ^{13}C edHSQC NMR spectrum (400 MHz, $CDCl_3$) of deceptionin (**7**).

Figure S14. ^{1}H, ^{13}C HMBC NMR spectrum (400 MHz, $CDCl_3$) of deceptionin (**7**).

Figure S15. ^{1}H, ^{1}H NOESY NMR spectrum (400 MHz, $CDCl_3$) of deceptionin (**7**).

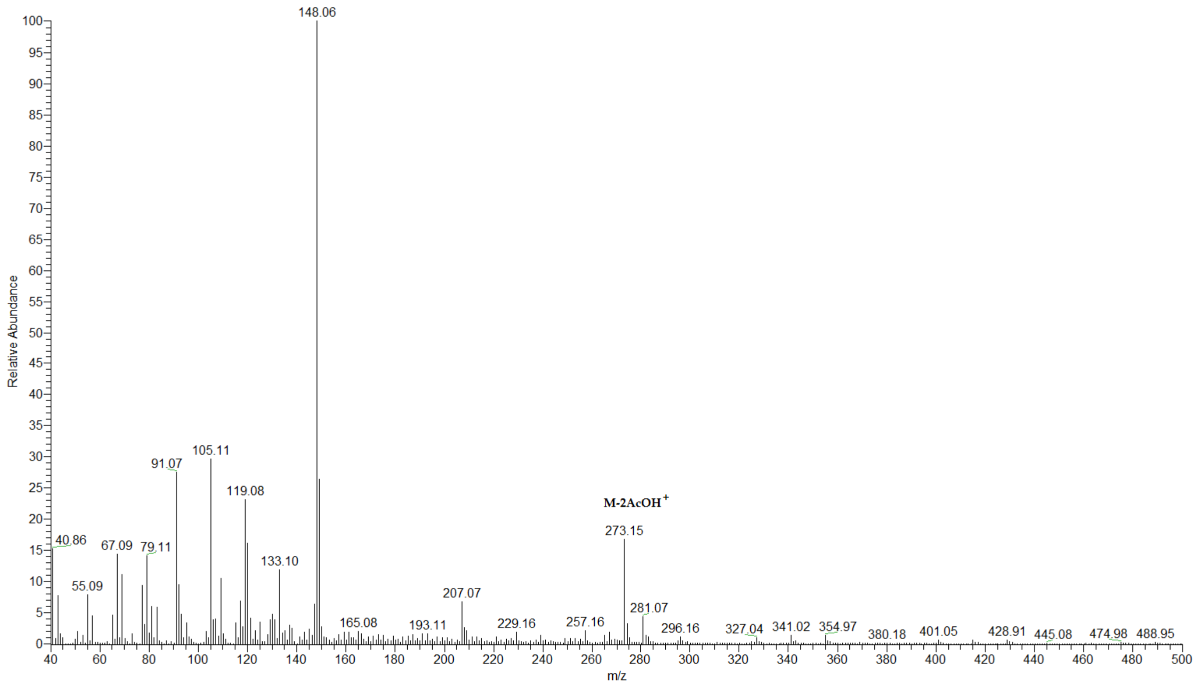

Figure S1. EIMS spectrum of 9,11-dihydrogracilin A (**1**).

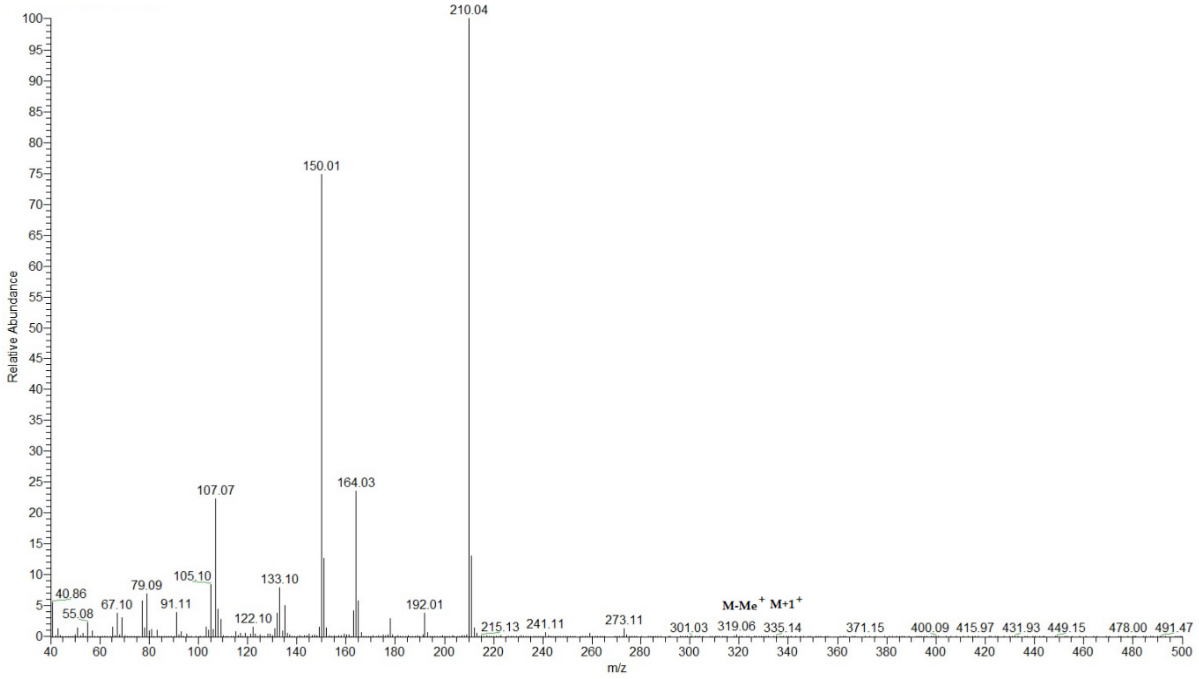

Figure S2. EIMS spectrum of compound **2**.

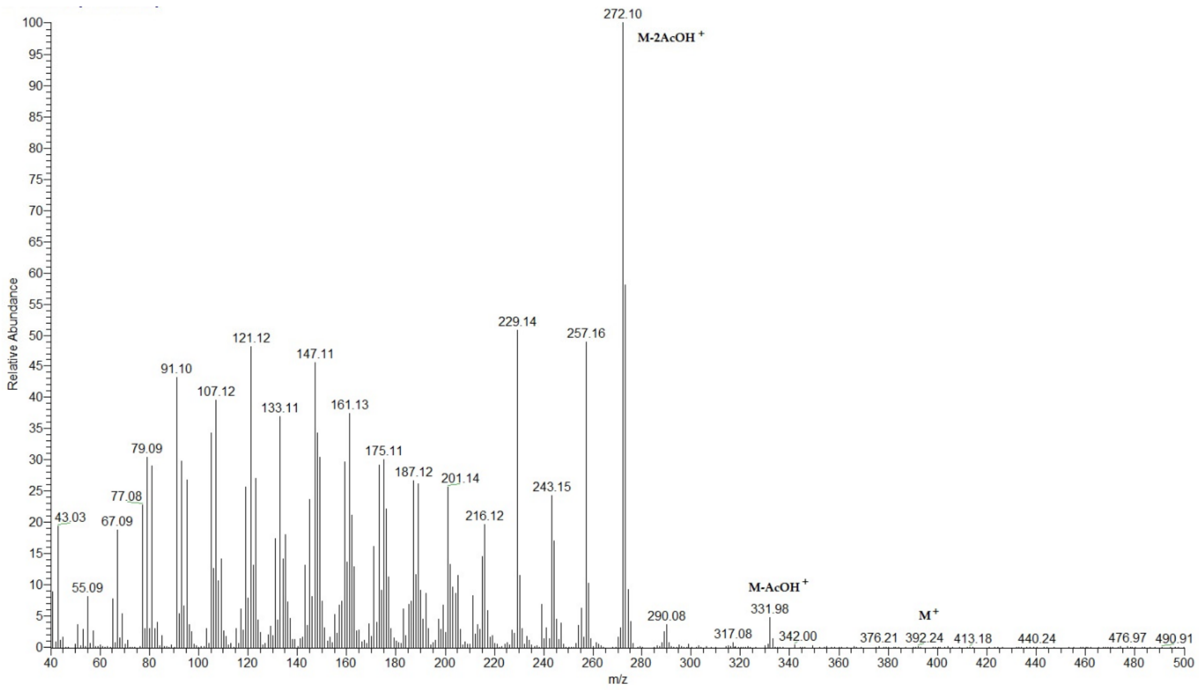

Figure S3. EIMS spectrum of compound **3.**

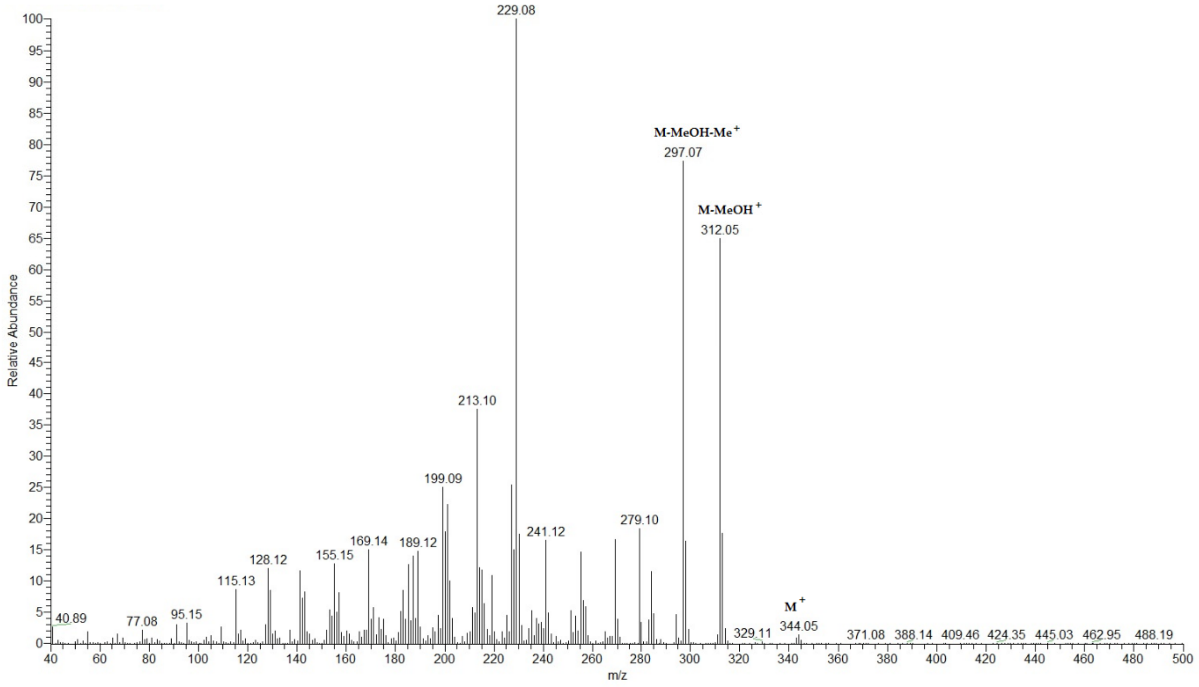

Figure S4. EIMS spectrum of membranolide (**4**).

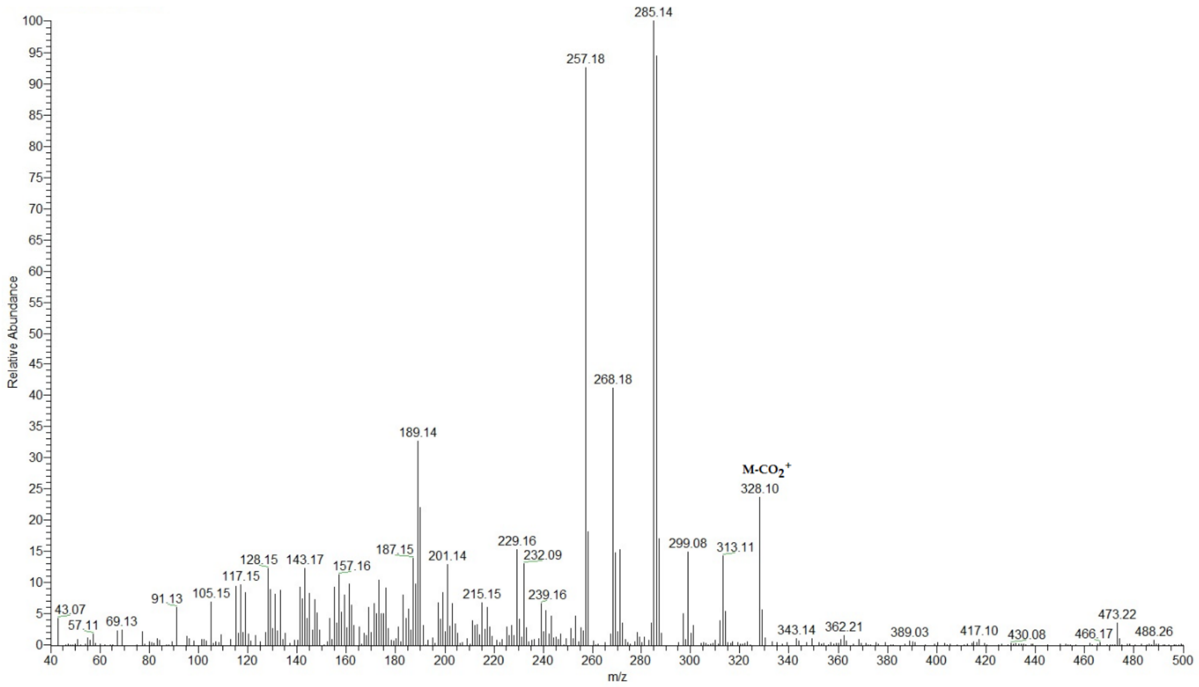

Figure S5. EIMS spectrum of aplysulphurin (**5**).

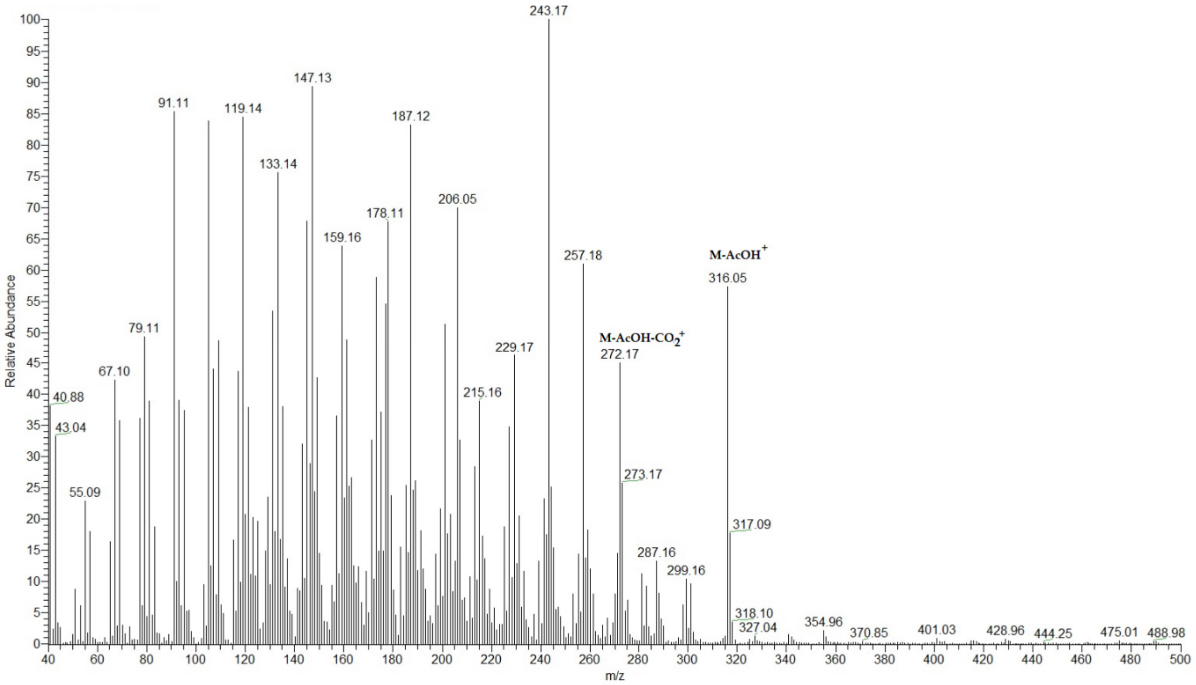

Figure S6. EIMS spectrum of tetrahydroaplysulphurin-1 (**6**)

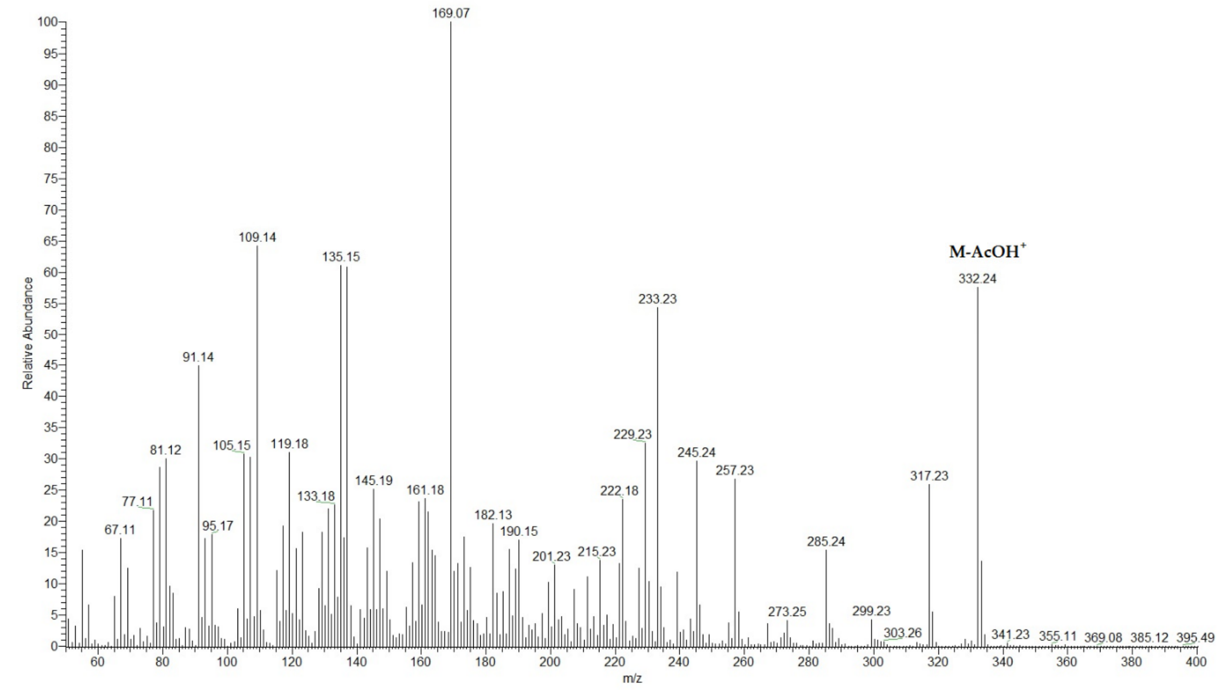

Figure S7. EIMS spectrum of deceptionin (**7**).

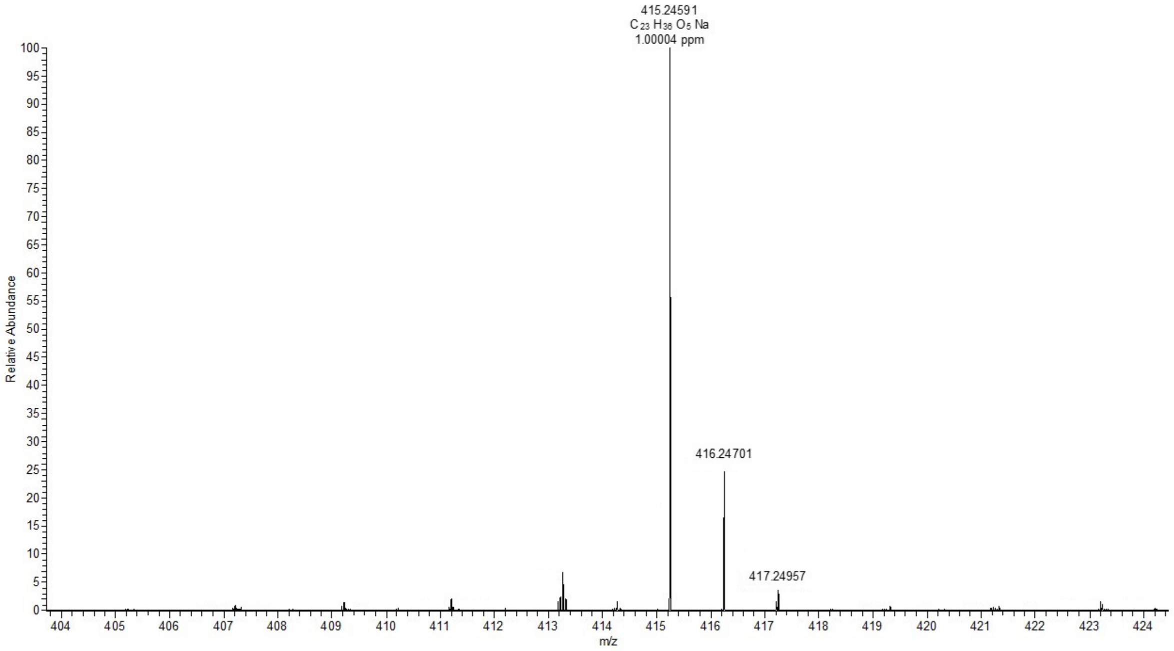

Figure S8. HRESI-MS of deceptionin (**7**).

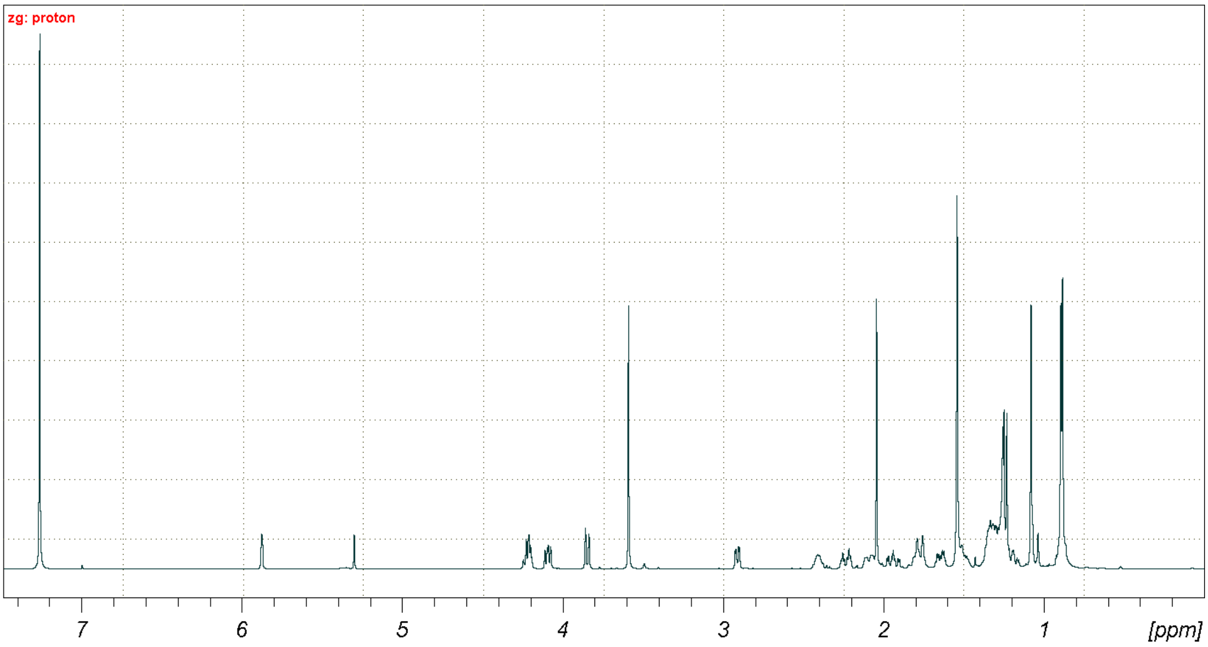

Figure S9. ^{1}H NMR spectrum (400 MHz, $CDCl_3$) of deceptionin (**7**).

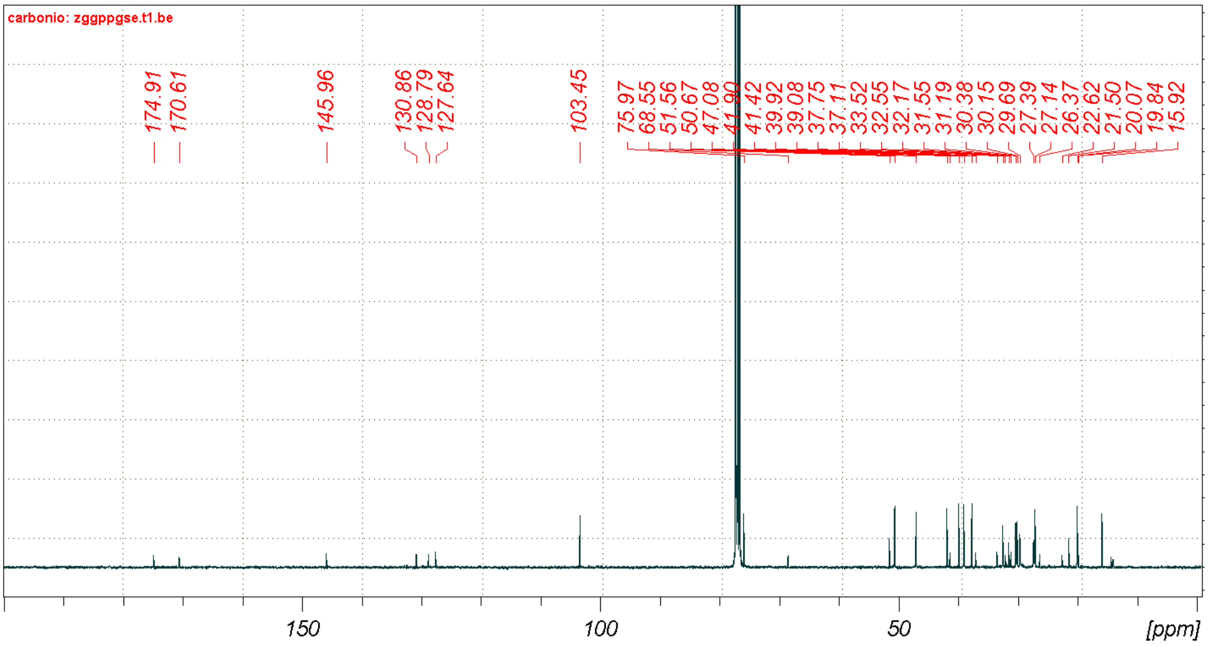

Figure S10. ^{13}C NMR spectrum (100 MHz, $CDCl_3$) of deceptionin (**7**).

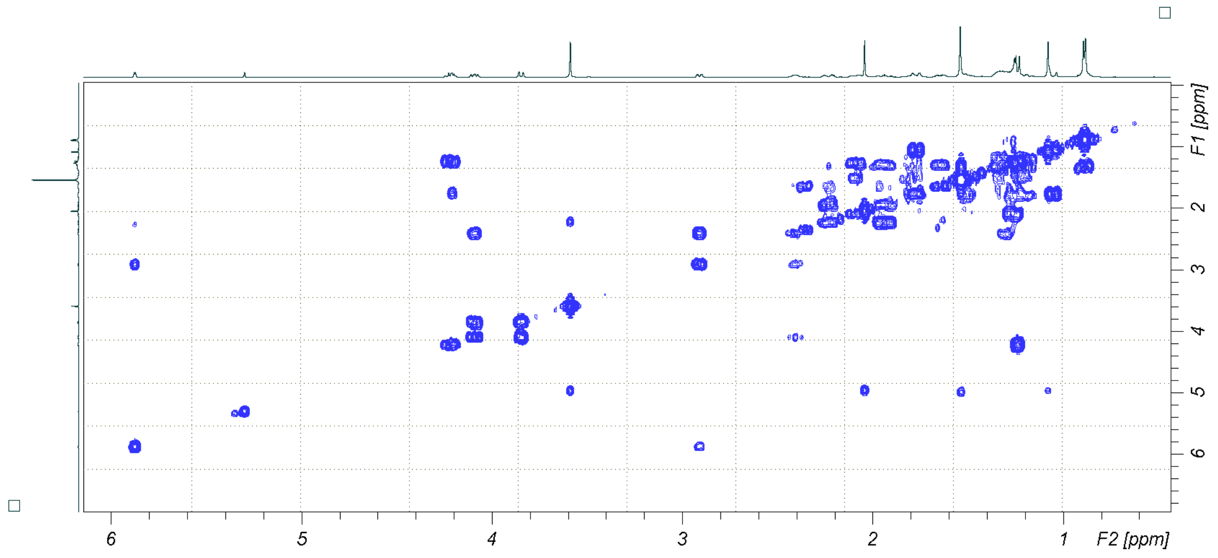

Figure S11. $^{1}H,^{1}H$ COSY NMR spectrum (400 MHz, $CDCl_3$) of deceptionin (**7**).

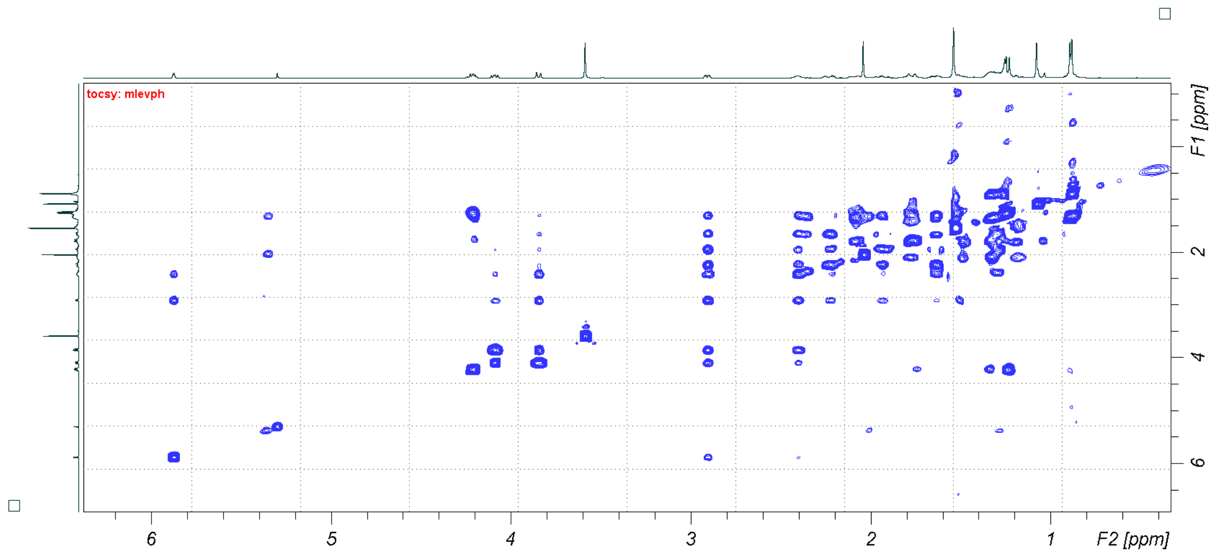

Figure S12. $^{1}H,^{1}H$ TOCSY NMR spectrum (400 MHz, $CDCl_3$) of deceptionin (**7**).

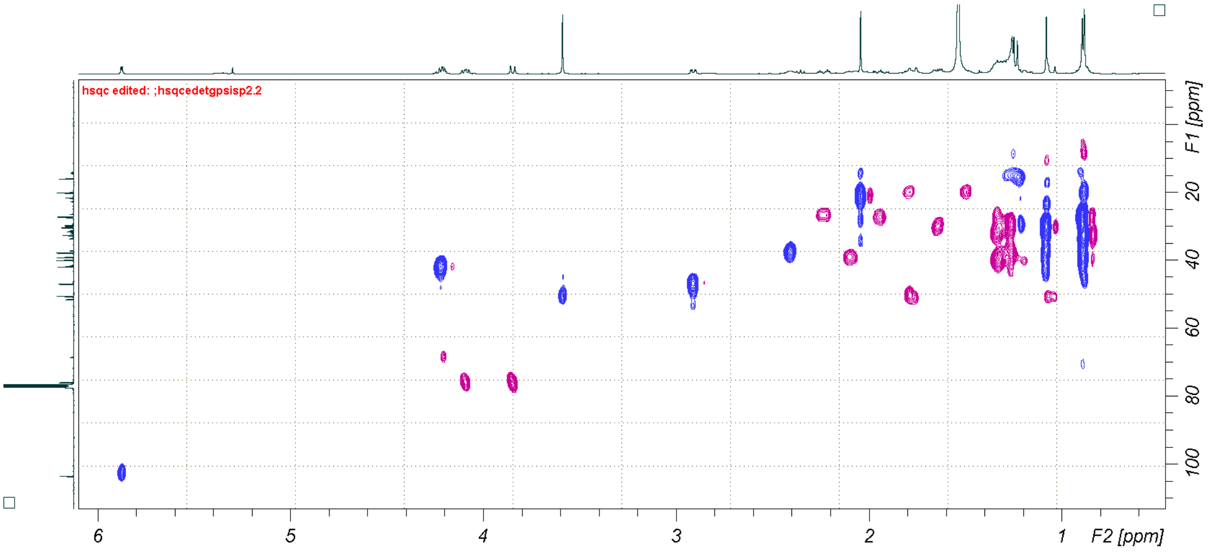

Figure S13. ^{1}H, ^{13}C edHSQC NMR spectrum (400 MHz, $CDCl_3$) of deceptionin (**7**).

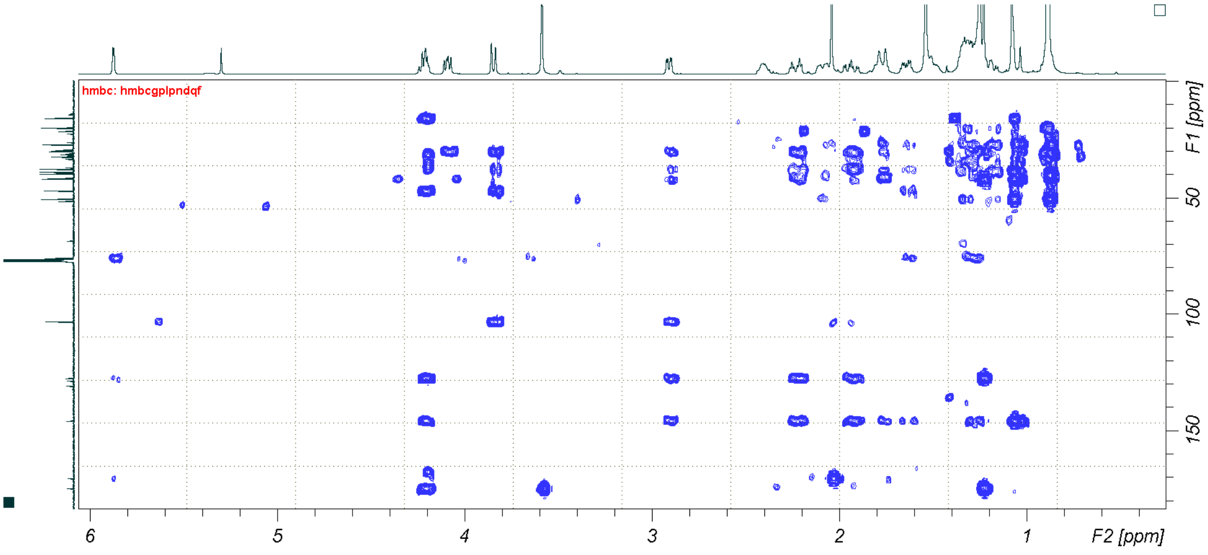

Figure S14. ^{1}H, ^{13}C HMBC NMR spectrum (400 MHz, $CDCl_3$) of deceptionin (**7**).

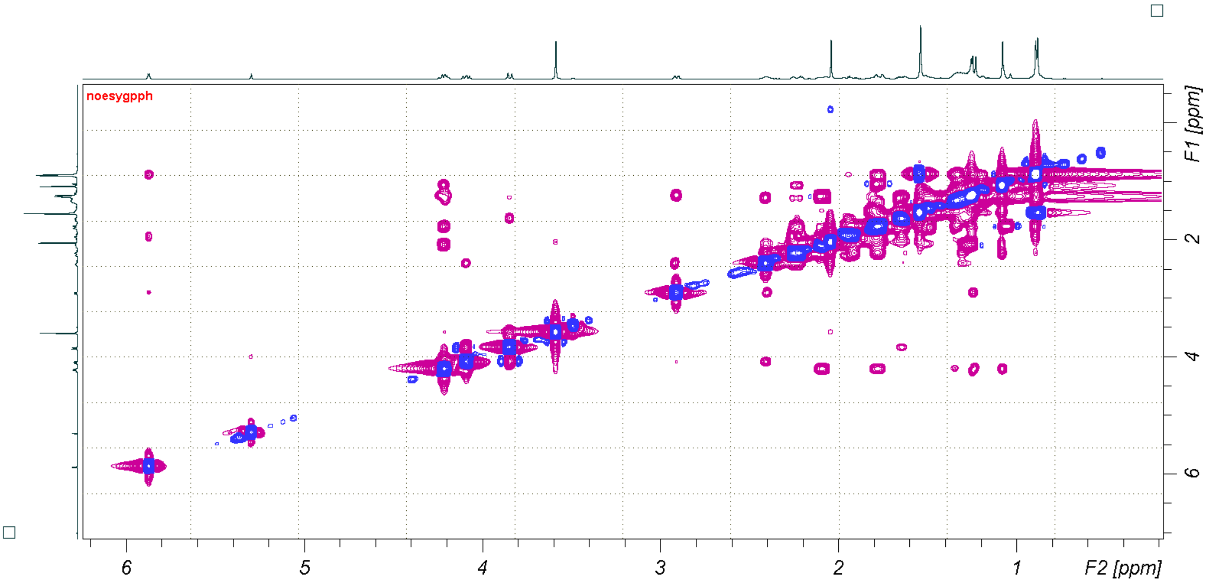

Figure S15. ^{1}H, ^{1}H NOESY NMR spectrum (400 MHz, $CDCl_3$) of deceptionin (**7**).
